# Mullerian adenosarcoma of the uterus with MASO: a case report of cervical adenosarcoma in a young female that never had sexual behavior

**DOI:** 10.1186/s12905-024-03140-w

**Published:** 2024-05-23

**Authors:** Jia-Wei Chen, Ya-Jie Huang, Ling-Lu Wang, Jun-Jiang Liu, Ming-Mei Shi, Na Li

**Affiliations:** 1 Department of Obstetrics and Gynecology, The Second Affiliated Hospital of Zunyi Medical University, The intersection of Xinlong Avenue and Xinpu Avenue, Honghuagang District, Zunyi, Guizhou Province 563000 China; 2 Department of Obstetrics and Gynecology, The Sixth People’s Hospital of Chengdu, Chengdu, Sichuan 610051 China

**Keywords:** Asexual women, Mullerian adenosarcoma, Sarcomatous overgrowth, Cervical tumor, Uterine adenosarcoma

## Abstract

**Background:**

Cervical mullerian adenosarcoma is a rare uterine sarcoma, especially in young women. Its pathological features are low-grade malignant tumors with bidirectional differentiation, and the degree of malignancy is similar to that of low-grade endometrial stromal sarcoma. This paper reports the case of a young asexual patient who has been closely followed up after tumor resection and has not had any recurrences.

**Case presentation:**

A 20-year-old, young asexual woman was diagnosed with cervical mullerian adenosarcoma with sarcomatous overgrowth (MASO). Cervical tumor resection was performed after admission, and the resection margin was negative. After the operation, she refused to undergo secondary surgery due to fertility requirements and did not receive adjuvant treatment. The patient was closely followed up after the operation and has not yet relapsed.

**Conclusion:**

A young woman with cervical MASO did not receive adjuvant treatment after cervical tumor resection. For women with fertility requirements, close follow-ups should be conducted after the operation to guard against tumor recurrence and radical tumor resection should be performed as early as possible after the patient no longer requires their fertility.

## Background

Mullerian adenosarcoma (MA) is a rare type of uterine sarcoma. It is a mixed tumor composed of benign epithelial and malignant stromal components [1]. It usually occurs in the body of the uterus and rarely in the ovary or cervix [2]. It has several high-risk factors, including mullerian adenosarcoma with sarcomatous overgrowth (MASO), high mitotic rate, myometrial invasion, and extrauterine metastasis [3]. This paper reports a case of a young person with MASO at the cervix. The paper reviews the relevant literature, thereby exploring the clinical characteristics, diagnosis, treatment, and prognosis of cervical MA and providing a clinical reference for treating this rare case.

## Clinical data

**Fig. 1:**
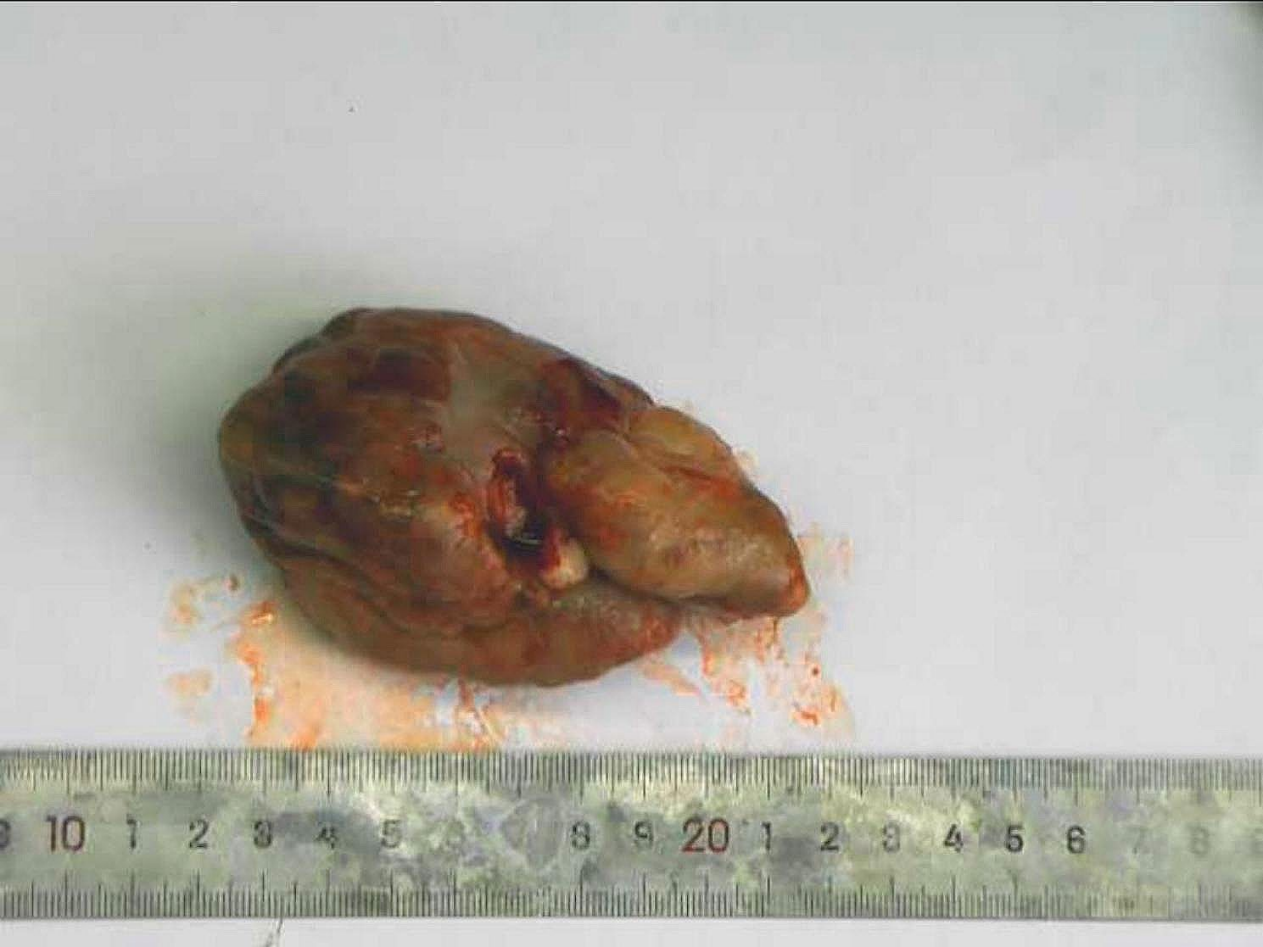
Picture for tumor gross examination

The young patient was a 20-year-old asexual female. She was admitted to the hospital for three months after detecting a vulval tumor. The patient had previously been in good health. A vulva mass had been palpated three months before the patient’s admission, and it was approximately the size of the thumb and was continuously enlarging, with mucoid and bloody secretions. The results of the gynecological examination showed it was a solid mass, approximately 3 × 6 cm in size, found at the vaginal orifice. The mass was connected to the body through a pedicle, its root was located in the cervix, and the vagina was consequently closed by the space the mass was occupying. The biochemical blood tests showed that the human chorionic gonadotropin reading was negative. The results showed that the levels of alpha-fetoprotein, cancer antigen 125, carbohydrate antigen 19 − 9, human epididymis protein 4, and carcinoembryonic antigen (CEA) were normal. A Doppler ultrasound by color found no obvious abnormalities in the uterus and double appendages. Screening for HPV (human papillomavirus) showed negative. Cervical tumor resection was performed on February 24, 2022. During the operation, pus spots were found on the surface of the tumor, and the odor was bad. The tumor’s pedicle was connected to the left side of the cervix, and the tumor prolapsed from the vaginal orifice (Fig. 1). A subsequent frozen section pathological procedure suggested the lesions were benign.

**Fig. 2:**
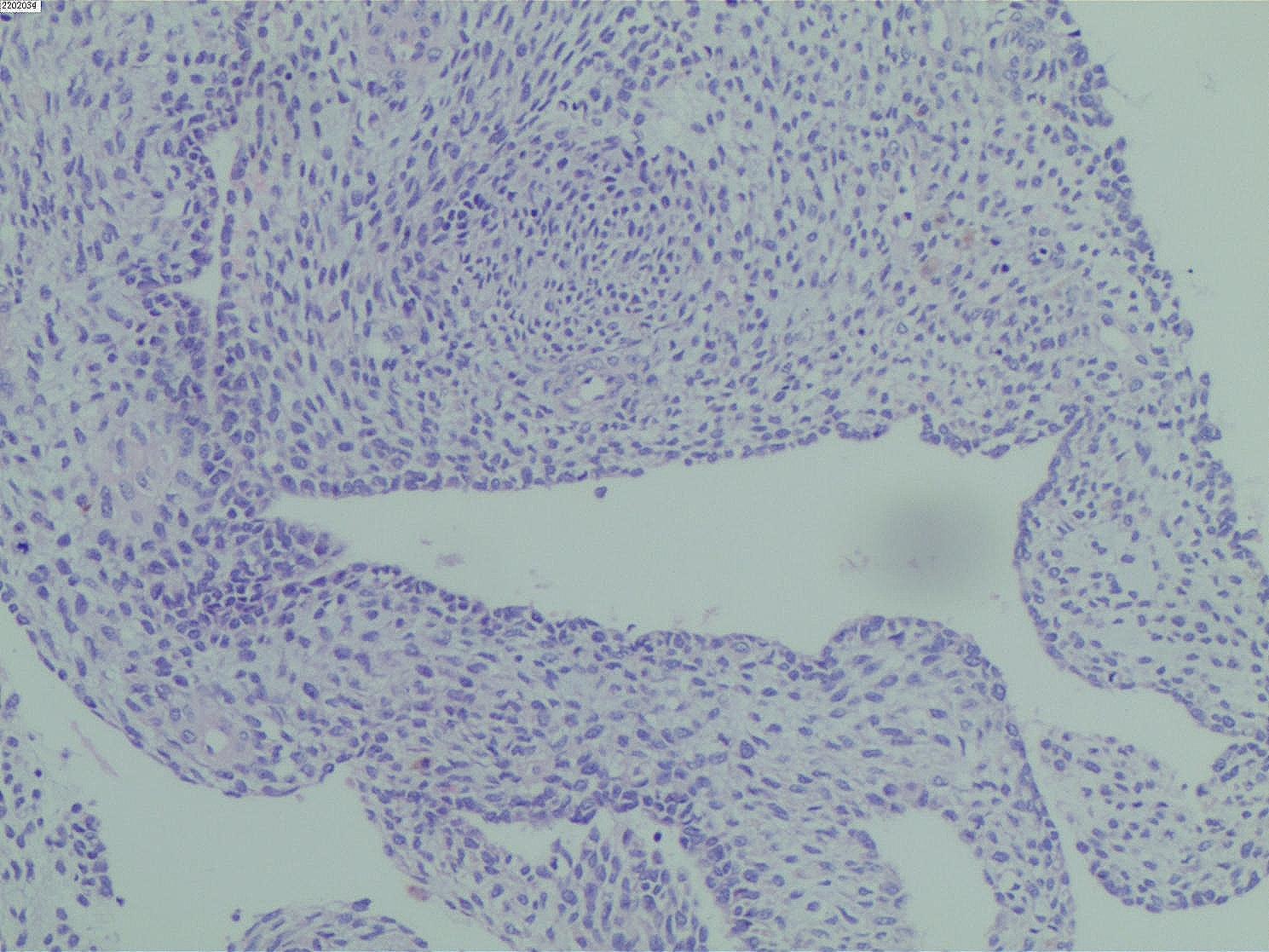
Tumor microscopic examination picture (H&E staining 200×)

**Fig. 3:**
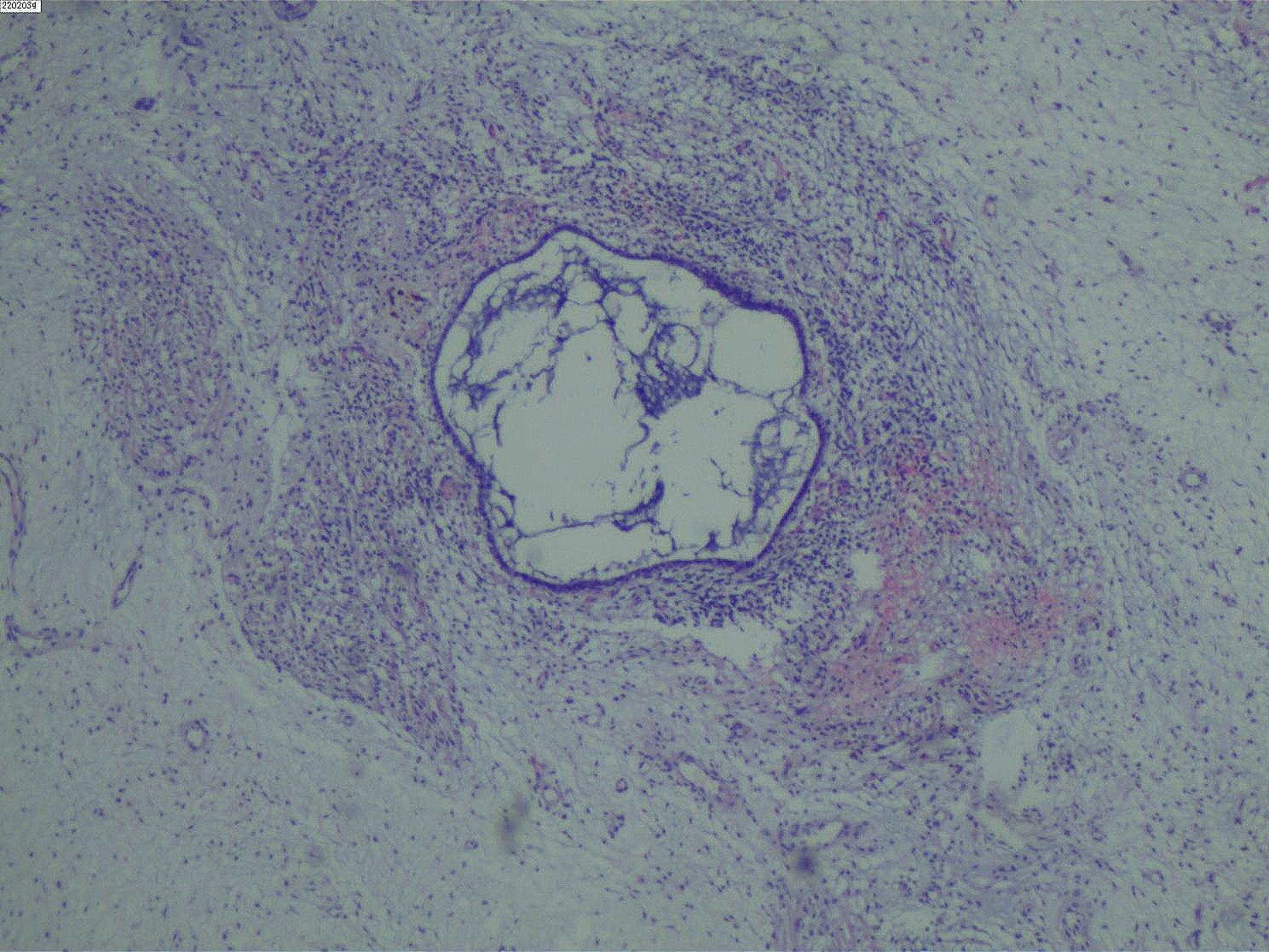
Tumor microscopic examination picture (H&E staining 100×)

**Fig. 4:**
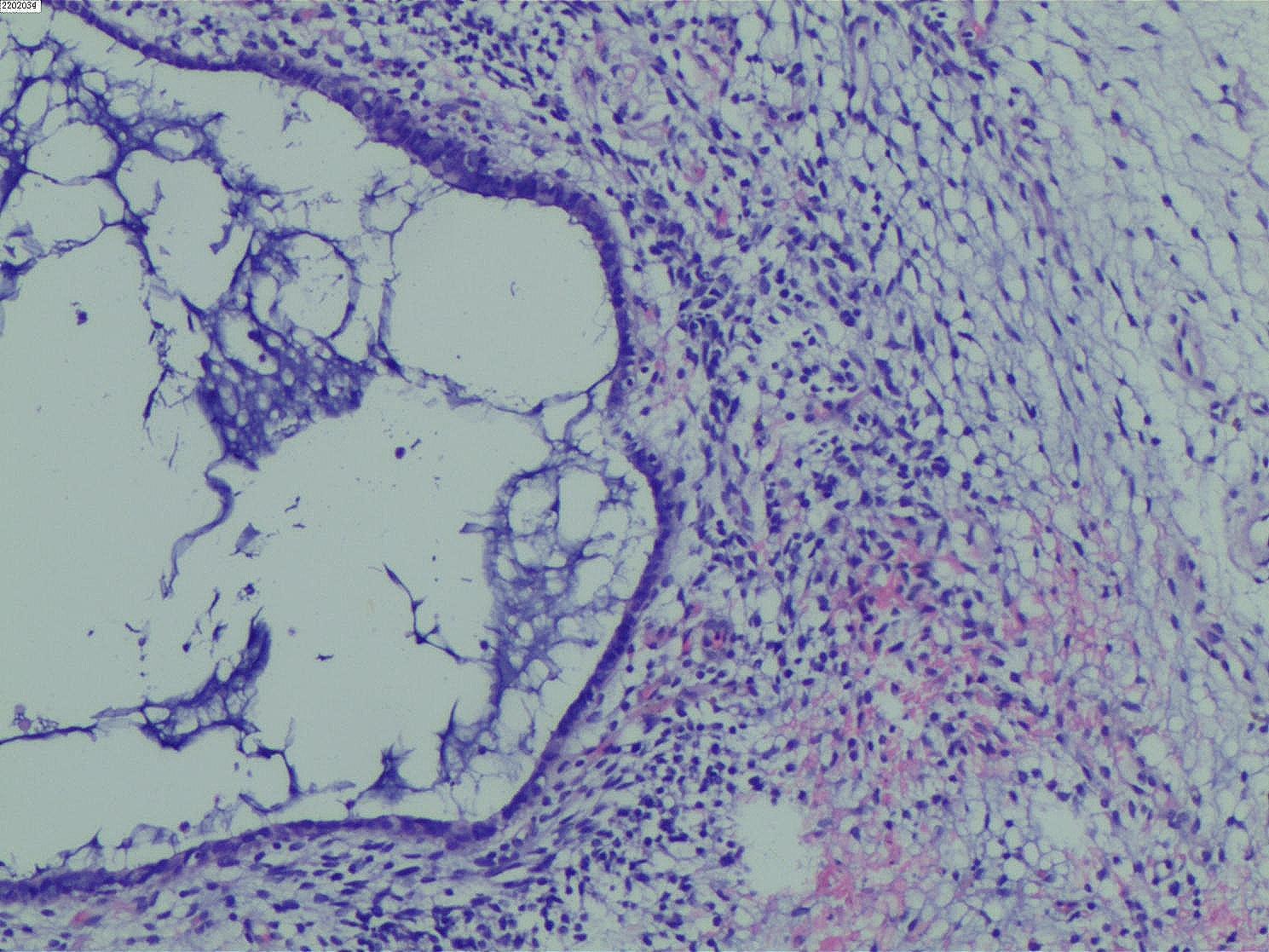
Tumor microscopic examination picture (H&E staining 200×)

After being reviewed by three senior pathologists in our hospital, the paraffin pathological examination results (Figs. 2 and 3, and 4) indicated that the tumor was a cervical MA. The sarcomatous component area showed sarcomatous overgrowth with no heterogenic component. No tissue involvement was found at the resection margin, and tumor tissue involvement was found at the base. The immunohistochemical results displayed positive readings for vimentin, creatine kinase (CK) in the epithelium, and cytokeratin 7 in the glandular epithelium. There were negative readings for desmin, myogenin, myoblast determination protein 1 (MyoD1), CEA, cluster of differentiation 10, estrogen receptor (ER), and progesterone receptor (PR). The antigen KI67 (Ki-67) score was 40%. Combined with a pathological diagnosis, it was diagnosed as the International Federation of Obstetrics and Gynecology (FIGO) stage IA of cervical MASO. Because the patient was young and had fertility requirements, she refused the second operation and postoperative adjuvant treatment. The patient was closely followed up, and no recurrence occurred for more than six months after the operation.

## Discussion

Uterine MA is a rare uterine sarcoma that occurs primarily in postmenopausal or premenopausal women. The incidence is low in young women, and ethnic and genetic differences may contribute to differences in the age of onset [4]. When the sarcomatous tissue component accounts for more than 25% of the total tumor volume, it is called a MASO [2]. Most adenosarcomas originate from the endometrium (including the lower uterine segment), and a small number of them originate from the endocervix (5–10%) and extrauterine sites [5]. The most common sites were the uterus (71%) and ovaries (15%), and rarely originated in the cervix (2%). Tumors outside the uterus are more common in adolescents and young women [6]. This is a rare case of a 20-year-old woman, whose tumor originated in the cervix. After a resection, 25–30% of patients relapse within five years. Recurrences are more common in patients with myometrial invasion and overgrowth of sarcomatous elements [7]. This patient was complicated with excessive growth of sarcoma as a high-risk factor and should be followed up closely after surgery.

### Pathology

Uterine MA belongs to the mixed epithelial-mesenchymal tumor category and is a low-grade malignant tumor with bidirectional differentiation. The epithelial glandular component is benign, and the mesenchymal component is malignant, and its degree of malignancy is similar to that of low-grade endometrial stromal sarcoma [8]. Its typical pathological feature is a sleeve-like shape formed by sarcomatous elements surrounding the glands [9]. A study concluded that adenosarcoma might be associated with endometriosis and adenomyosis [10]. Clement et al. considered those who met one or more of the following criteria to be diagnosed [11]: ① ten High Power Fields (HPFs) had two or more mitotic figures (MFs); ② Significant stromal cell; ③ Significant mesenchymal cell atypia.

Some studies have found that adenomyosis and endometriosis may be associated with the occurrence of Müllerian adenosarcoma, and endometriosis bleeding induces persistent oxidative stress and DNA damage, which may be one of the influencing factors of the disease [12, 13]. For cervix-derived Müllerian adenosarcoma, differs from most intracervical lesions, although the persistence of HPV is thought to be one of the major risk factors for the development of intracervical lesions and recurrence after treatment [14, 15]. However, the disease may be a non-HPV-related disease. Regarding MAs of cervical origin, a study involving 10 patients showed no high-risk human-based tumor viruses were detected by cervical screening [1, 16]. High-risk human papillomaviruses have also not been detected in cases with sarcomatous overgrowth [3]. HPV infection was also not detected in this patient. This would suggest that high-risk human-based tumor viruses are less likely to be involved in the occurrence and development of such tumors. For such non-HPV-related diseases, patients should not rely solely on a negative HPV test to ignore the possibility of cervical lesions, especially young women, who should pay more attention to the importance of gynecological examination.

In applying immune-related molecules, the epithelial components of tumors express epithelial markers such as CK and epithelial membrane antigen. Cluster of differentiation 10, WT1, ER, and PR are highly expressed in the sarcomatous component, among which ER and PR positivity is detected in about 50–80% of adenosarcomas. In the case of sarcomatous overgrowth, it exhibits dedifferentiation of the mesenchymal component, and the expression of these markers is usually reduced [17]. This patient was complicated with sarcoma overgrowth, and immunohistochemical indicators such as CD10, ER, and PR were negative. The Ki-67 score indicates the degree of proliferation as an index. The higher the proliferation activity of the tumor is, the higher the expression level of Ki-67. If the patient is accompanied by heterologous sarcoma components, such as rhabdomyosarcoma components with positive expressions of Desmin, Myogenin, and MyoD1, the risk of recurrence may increase [18]. However, whether the negative results of Desmin, Myogenin, and MyoD1 in this patient suggest that the risk of recurrence is small, further follow-up is needed to determine the prognosis. Changes in the tumor protein 53 (p53) pathway are common in high-grade adenosarcomas, regardless of the presence or absence of sarcoma overgrowth. The over-expression of p53 is associated with tumor recurrence and metastasis [19]. In the case presented in this paper, it was diagnosed as cervical MASO, as it had strong expression of epithelial components but had no expression of heterologous sarcoma components. The recurrence risk and prognosis require further follow-up.

Mullerian adenosarcoma should also be differentiated from other diseases. For example, endometrial stromal sarcoma is composed of a single endometrial stromal cell with an infiltrating growth border and no epithelial component. The diagnosis can only be made after complete resection of the tumor by sampling multiple sites to determine whether there is an epithelial component. Uterine carcinosarcoma: a mixed tumor composed mainly of malignant glands and malignant mesenchymal components. Current studies suggest that the invasion behavior of this tumor is determined by the composition of malignant epithelial components, while the malignancy of MA is mainly composed of mesenchymal components. Endometrial polyp: Pathological lack of interstitial atypia, interstitial cell proliferation, peri glandular cuff changes, and muscular infiltration of morphological features; Condyloma acuminatum: the upper part of the spinous layer can have obvious vacuolar degeneration, HPV testing is generally positive.

### Diagnosis and treatment

The most common clinical manifestation of an MA is vaginal bleeding. Few patients receive treatment due to pelvic pain, abdominal mass, or abnormal vaginal secretions [20]. A gynecological examination may reveal a prolapsed mass from the cervix or a palpable pelvic mass. An ultrasound should show an enlarged uterus and solid intrauterine mass that is mostly hypoechoic. Using color Doppler flow imaging (CDFI) shows that the blood flow signal of uterine adenosarcoma is mostly solid, cystic, or mixed cystic–solid. An adenosarcoma outside the uterus is often cystic hyperechoic [21]. In a magnetic resonance examination, uterine adenosarcoma is mostly displayed as isointense nodules on T1-weighted imaging and as mixed signals on T2-weighted imaging. Small hyperintense signals of cystic foci with abundant blood supplies are seen in the tumor [22]. Due to the lack of specificity in clinical symptoms, signs, and supplementary examinations, MAs are easily misdiagnosed preoperatively. Because of its bidirectional differentiation, there may be differences between the frozen section pathological procedure and the paraffin pathological results. The exact diagnosis relies on the paraffin pathological diagnosis after tumor resection.

Surgical treatment is preferred for uterine MA, and a total hysterectomy and bilateral adnexectomy are usually carried out [23]. Surgical staging has an important impact on prognosis. Maintaining negative resection margins is the focus of surgery. Mullerian adenosarcoma generally has a good prognosis with less distant metastasis, but local recurrence is possible. The risk factors for uterine adenosarcoma mainly include age, sarcomatous overgrowth, myometrial invasion, lymph node metastasis, and vascular invasion [24]. In the case of sarcomatous overgrowth, the overall probability of survival is significantly reduced [25]. For young women with reproductive requirements, one study proposes that, if the patient is at FIGO stage IA, that is, the tumor is confined to the endometrium or cervical canal [26], and there are no risk factors such as deep myometrial invasion and sarcomatous overgrowth, tumor resection alone may be enough, and the patient should be closely followed-up and rechecked after the operation. When a patient no longer needs to be fertile, a total hysterectomy and bilateral adnexectomy should be carried out [27]. The two-year survival rate for patients with uterine adenosarcoma is 100%; in contrast, the two-year survival rate for patients with sarcomatous overgrowth is only 20% [28–30]. In this case, the patient had sarcoma overgrowth but refused radical surgery and postoperative chemoradiotherapy, and should be closely followed up to be vigilant for tumor recurrence. The resection of cervical lesions increases the risk of preterm birth and preterm premature rupture of membranes [31]. However, there have been case reports of good pregnancy outcomes in patients after cervical tumor resection [27, 32]. This patient does not currently have a pregnancy plan, and further follow-up is needed for studies on the effects of fertility in this patient. In addition, we summarize some of the reported Müllerian adenosarcomas.

**Table:** 

References	Age (Years)	Follow-up time	High-risk features	Follow-up
Pedram Fadav et al. [33]	63	9 months	Without risk factor	No signs of recurrence
Qiyue et al. [34]	38	15 months	Without risk factor	No signs of recurrence
Garcia-Mendoza et al. [2]	46	3 months	Sarcomatous overgrowth with heterologous elements of grade 2 chondrosarcoma	Palliative treatment
Goh C et al. [4]	21	8 years	Without risk factor	Recurrence
D.D. Dowding [35]	24	19 months	Arcomatous overgrowth	No signs of recurrence

There is still no consensus on postoperative adjuvant therapy for adenosarcomas. A study concluded that adjuvant chemotherapy was feasible for patients with high-risk factors to reduce the risk of recurrences and improve long-term survival [36]. Now the chemotherapy regimen of doxorubicin combined with ifosfamide is more commonly used [37]. Because ER/PR expression is lower in tumors with sarcomatous overgrowth, the application of hormonal therapy should be limited. Although some studies have suggested that hormone therapy should be considered if ER/PR is positive, there is still a lack of clinical and research-based evidence for hormone therapy in treating adenosarcoma [38, 39].

## Conclusion

In summary, this study introduced a case of cervical Müllerian adenosarcoma, which is a sarcomatous overgrowth, which was rare in young females without sexual behavior never and was easily misdiagnosed as uterine polyps or ovulatory abnormal uterine bleeding at the initial diagnosis, and the importance of gynecological examination should be emphasized. Close follow-up of fertility-preserving patients to further understand the impact on pregnancy. For patients with fertility requirements, complete resection of the tumor to achieve a negative resection margin is the basic requirement of surgery. The existing clinical guidelines for the treatment of young patients, especially those with fertility requirements, are unclear, and close follow-up of the tumor and fertility outcome is required. In the future, more clinical studies are needed to further provide evidence and improve the treatment strategy for this disease.

## Data Availability

The data that support the findings of this study are available from the corresponding author, upon reasonable request.

## Abbreviations

MA: Mullerian adenosarcoma
MASO: Mullerian adenosarcoma with sarcomatous overgrowth
ER: estrogen receptor
PR: progesterone receptor
HPF: high power field
CDFI: color Doppler flow imaging
